# CFTR-rich ionocytes mediate chloride absorption across airway epithelia

**DOI:** 10.1172/JCI171268

**Published:** 2023-10-16

**Authors:** Lei Lei, Soumba Traore, Guillermo S. Romano Ibarra, Philip H. Karp, Tayyab Rehman, David K. Meyerholz, Joseph Zabner, David A. Stoltz, Patrick L. Sinn, Michael J. Welsh, Paul B. McCray, Ian M. Thornell

**Affiliations:** 1 Stead Family Department of Pediatrics and Pappajohn Biomedical Institute, Roy J. and Lucille A. Carver College of Medicine,; 2 Department of Internal Medicine and Pappajohn Biomedical Institute, Roy J. and Lucille A. Carver College of Medicine,; 3 Howard Hughes Medical Institute,; 4 Department of Pathology, Roy J. and Lucille A. Carver College of Medicine,; 5 Department of Biomedical Engineering,; 6 Department of Molecular Physiology and Biophysics, Roy J. and Lucille A. Carver College of Medicine,; 7 Department of Microbiology and Immunology, Roy J. and Lucille A. Carver College of Medicine, University of Iowa, Iowa City, Iowa, USA.

**Keywords:** Cell Biology, Pulmonology, Chloride channels, Epithelial transport of ions and water, Ion channels

## Abstract

The volume and composition of a thin layer of liquid covering the airway surface defend the lung from inhaled pathogens and debris. Airway epithelia secrete Cl^–^ into the airway surface liquid through cystic fibrosis transmembrane conductance regulator (CFTR) channels, thereby increasing the volume of airway surface liquid. The discovery that pulmonary ionocytes contain high levels of CFTR led us to predict that ionocytes drive secretion. However, we found the opposite. Elevating ionocyte abundance increased liquid absorption, whereas reducing ionocyte abundance increased secretion. In contrast to other airway epithelial cells, ionocytes contained barttin/Cl^–^ channels in their basolateral membrane. Disrupting barttin/Cl^–^ channel function impaired liquid absorption, and overexpressing barttin/Cl^–^ channels increased absorption. Together, apical CFTR and basolateral barttin/Cl^–^ channels provide an electrically conductive pathway for Cl^–^ flow through ionocytes, and the transepithelial voltage generated by apical Na^+^ channels drives absorption. These findings indicate that ionocytes mediate liquid absorption, and secretory cells mediate liquid secretion. Segregating these counteracting activities to distinct cell types enables epithelia to precisely control the airway surface. Moreover, the divergent role of CFTR in ionocytes and secretory cells suggests that cystic fibrosis disrupts both liquid secretion and absorption.

## Introduction

Pulmonary ionocytes are a rare cell type accounting for approximately 1% of airway epithelial cells. Ionocytes have attracted attention because, although rare, in airway epithelia they contain about half of all the mRNA transcripts for the cystic fibrosis transmembrane conductance regulator (CFTR) anion channel (1–7), and mutations in the gene encoding CFTR cause cystic fibrosis (CF). Secretory cells contain the second highest abundance of CFTR transcripts — about one-tenth the number of CFTR transcripts as an ionocyte — but comprise more than 10% of epithelial cells.

Previous studies of airway epithelia indicate that cAMP-dependent phosphorylation opens apical membrane CFTR anion channels, stimulates transepithelial Cl^–^ secretion, and water follows through abundant aquaporin channels (8, 9). Loss of CFTR function in CF disrupts anion secretion and, thereby, respiratory host defenses, predisposing to bacterial infection and inflammation (10). This background led us to test the hypothesis that ionocytes mediate Cl^–^ and liquid secretion across airway epithelia.

## Results

### Ionocytes increase ASL absorption.

**Figure 1:**
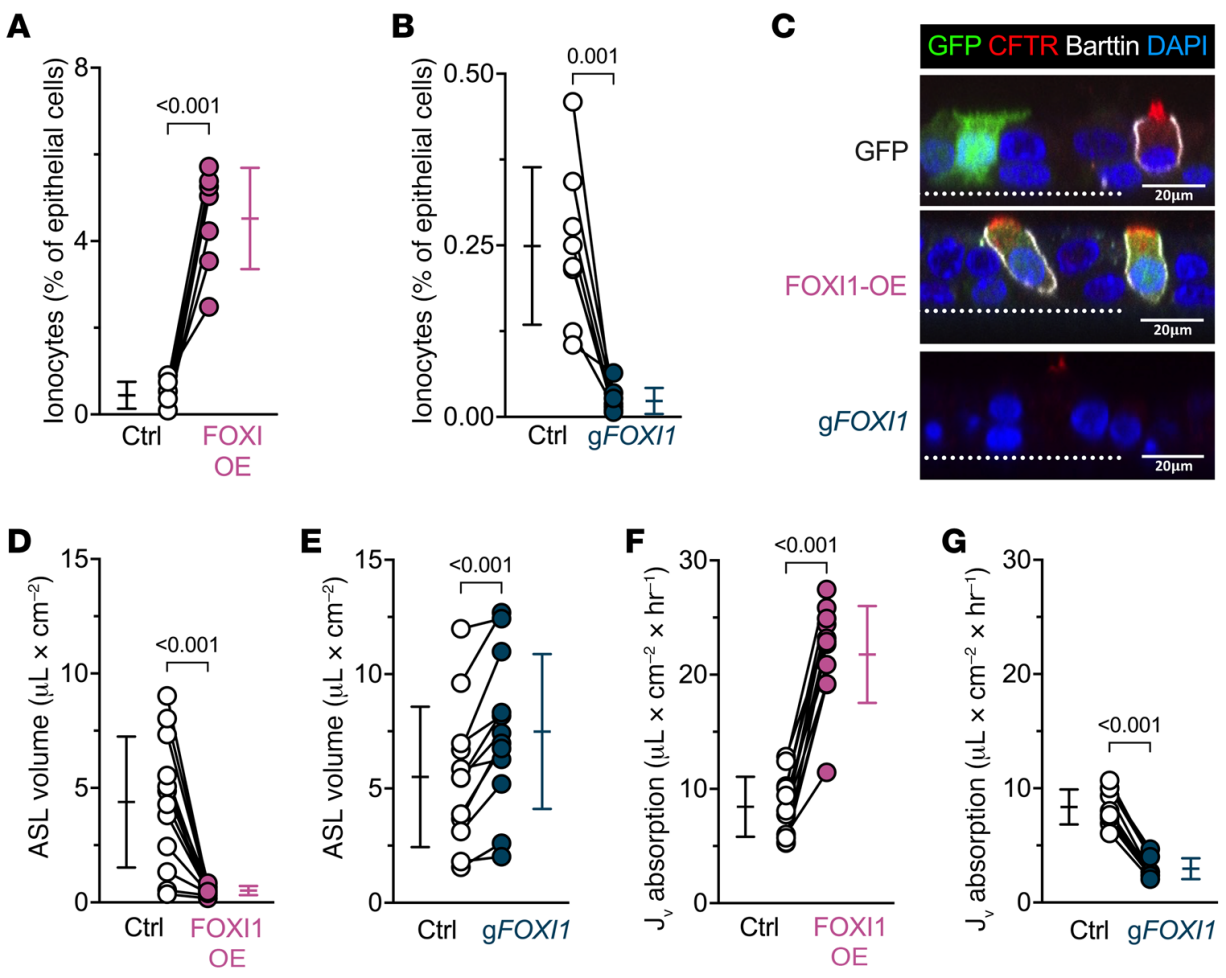
FOXI1 increases ionocyte abundance and decreases airway surface liquid (ASL) volume. Epithelia were transduced with a lentiviral vector expressing either GFP or a bicistronic construct expressing FOXI1 and GFP to generate and label ionocytes (FOXI1-OE), or they were electroporated with CRISPR/Cas9 and guide RNAs (gRNA) targeting *FOXI1* (g*FOXI1*) to decrease ionocyte abundance. (**A** and **B**) Flow cytometric analyses of epithelial cells expressing the ionocyte-specific protein barttin; *n* = 7–8 human donors. (**C**) Confocal images of human airway epithelia showing GFP (green), CFTR (red), barttin (white), and nuclei (blue). Dotted line represents the location of the culture filter. Scale bars: 20 μm. (**D** and **E**) ASL volume upon FOXI1-OE, g*FOXI1*, and their respective control epithelia; *n* = 12 human donors. (**F** and **G**) Liquid absorption rates for FOXI1-OE, g*FOXI1*, and their respective control epithelia; *n* = 10 to 12 human donors. Data points connected by a line represent paired experiments from a single human donor, graph depicts mean ± standard deviation, and *P* values obtained from paired, 2-sided Student’s *t* tests are presented within the figure.

We predicted that increasing the number of ionocytes would increase the volume of airway surface liquid (ASL). We transduced airway epithelial cell cultures with a lentivirus that overexpresses forkhead box I1 (FOXI1) to increase ionocyte abundance, an approach previously confirmed using single-cell RNA sequencing (2). After differentiation of the cultured airway epithelia, ionocyte abundance — indicated by barttin^+^ cells (11, 12) — increased (Figure 1, A and C, Supplemental Table 1, and Supplemental Figure 1A; supplemental material available online with this article; https://doi.org/10.1172/JCI171268DS1). But contrary to our expectations, ASL volume decreased (Figure 1D). Conversely, when we disrupted the *FOXI1* gene, which is required to form ionocytes from airway basal cells (1–3), ionocyte abundance decreased and ASL volume increased (Figure 1, B, C, and E, and Supplemental Figure 1B). As an additional test, we added saline to the apical surface and found that elevating ionocyte abundance increased liquid absorption, and depleting ionocyte numbers decreased liquid absorption (Figure 1, F and G). These findings indicate that rather than performing ASL secretion, ionocytes mediate ASL absorption.

**Figure 2:**
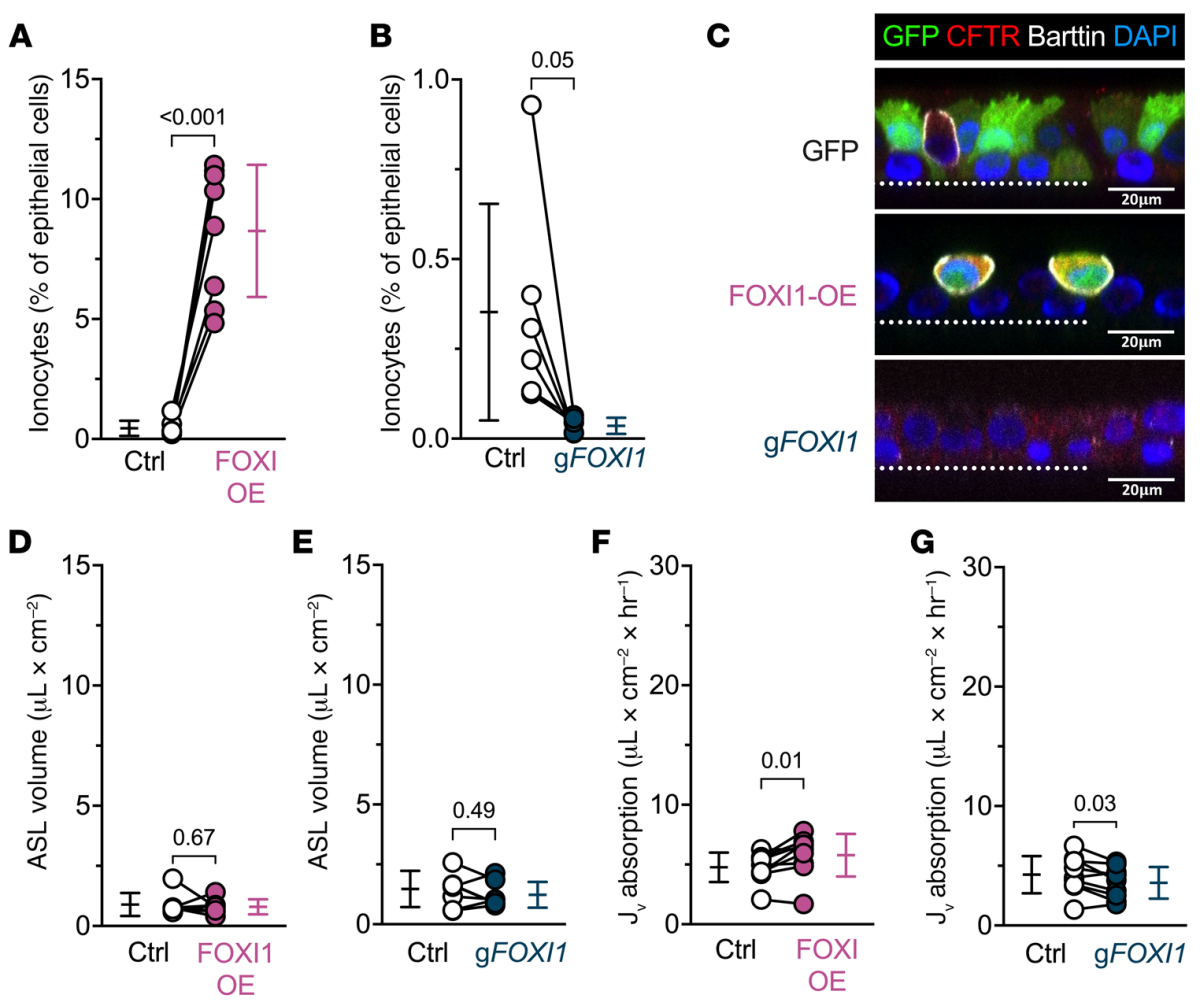
FOXI1 increases ionocyte abundance in cystic fibrosis epithelia, with nominal effects on the absorption of airway surface liquid (ASL). Cystic fibrosis (CF) epithelia were transduced with a lentiviral vector expressing either GFP or a bicistronic construct expressing FOXI1 and GFP to generate and label ionocytes (FOXI1-OE), or they were electroporated with CRISPR/Cas9 and guide RNAs (gRNA) targeting *FOXI1* (g*FOXI1*) to decrease ionocyte abundance. (**A** and **B**) Flow cytometric analyses of CF epithelial cells expressing the ionocyte-specific protein barttin; *n* = 6–8 human CF donors. (**C**) Confocal images of human CF airway epithelia showing GFP (green), CFTR (red), barttin (white), and nuclei (blue). Dotted line represents the location of the culture filter. Scale bars: 20 μm. (**D** and **E**) ASL volume upon FOXI1-OE, g*FOXI1*, and their respective control epithelia; *n* = 7 human CF donors. (**F** and **G**) Liquid absorption rates for FOXI1-OE, g*FOXI1*, and their respective control epithelia; *n* = 9 CF human donors. Data points connected by a line represent paired experiments from a single human donor, graph depicts mean ± standard deviation, and *P* values obtained from paired, 2-sided Student’s *t* tests are presented within the figure.

To test the contribution of CFTR to ionocyte function, we repeated the experiments in CF epithelia. Without CFTR function, increasing or decreasing ionocyte abundance failed to alter ASL volume (Figure 2). We observed a similar pattern for liquid absorption, although we observed a very small effect in some CF epithelia in the same direction as non-CF epithelia. These data indicate that CFTR channels play a key role in ionocyte liquid absorption.

**Figure 3:**
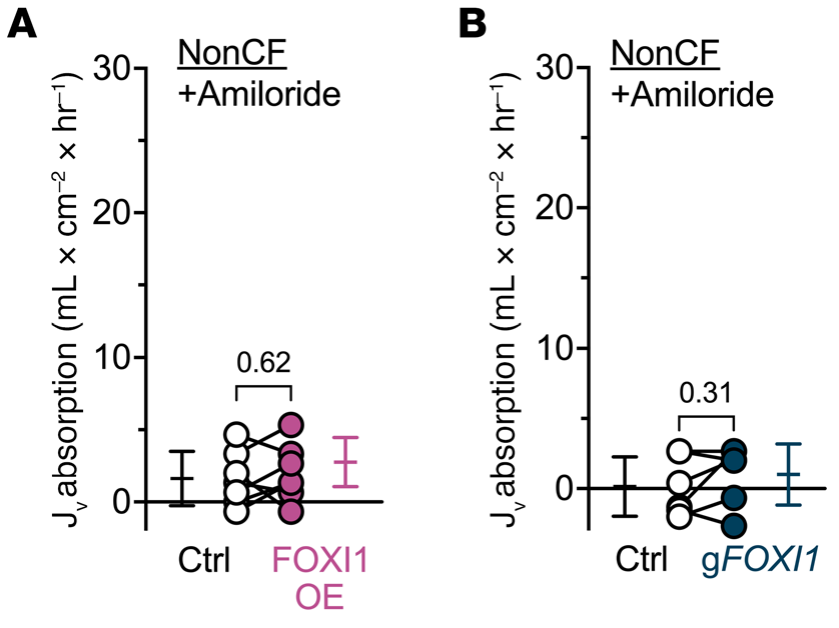
The effect of FOXI1 expression on liquid absorption requires ENaC activity. Liquid absorption assays with 100 μM amiloride included in the apical solution to evaluate the influence of ENaC on ionocyte-mediated liquid absorption. (**A**) Epithelia transduced with FOXI1 (FOXI-OE) to increase ionocyte abundance; *n* = 7 non-CF human donors. (**B**) Epithelia electroporated with CRISPR/Cas9 and guide RNAs (gRNA) targeting *FOXI1* (g*FOXI1*) to decrease ionocyte abundance; *n* = 6 non-CF human donors. Data points connected by a line represent paired experiments from a single human donor, graph depicts mean ± standard deviation, and *P* values obtained from paired, 2-sided Student’s *t* tests are presented within the figure.

Apical epithelial Na^+^ channels (ENaCs) control Na^+^ absorption across airway epithelia (13). Therefore, we added the ENaC inhibitor amiloride and found that it eliminated the effect of ionocytes on liquid absorption (Figure 3). These data suggest that the absorptive flow of Cl^–^ through ionocytes is driven by ENaC-dependent activity and thus the electrical gradient generated by Na^+^ absorption.

### Ionocytes increase current flow through airway epithelia.

**Figure 4:**
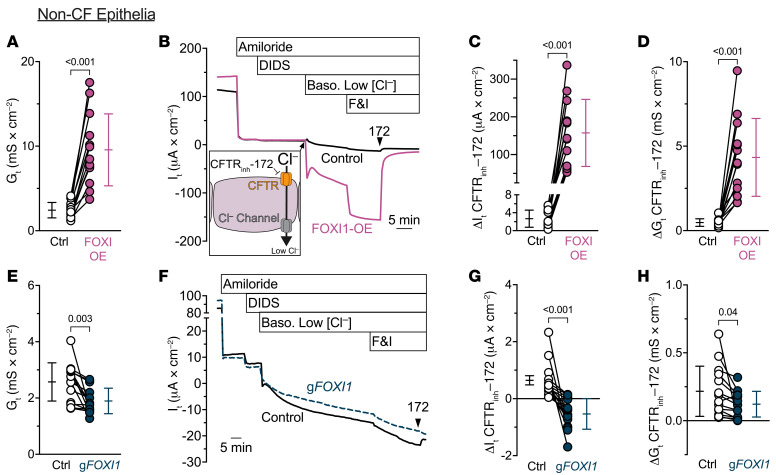
FOXI1 expression increases CFTR-dependent transepithelial current flow. (**A**–**D**) Increasing ionocyte abundance with FOXI1 overexpression increases transepithelial current flow; *n* = 13 human donors. (**A**) Transepithelial conductances (*G_t_*) obtained from epithelia bathed in symmetrical solutions. (**B**) Example transepithelial current (*I_t_*) recordings with the transepithelial voltage held at 0 mV. The basolateral [Cl^–^] was reduced to drive passive Cl^–^ current into the cell through apical CFTR channels and out of the cell through any basolateral Cl^–^ channels (depicted in inset). F&I, forskolin and 3-isobutyl-1-methylxanthine. (**C**) CFTR_inh_-172–sensitive *I_t_* data. (**D**) CFTR_inh_-172–sensitive *G_t_* data. (**E**–**H**) Decreasing ionocyte abundance by *FOXI1* disruption decreases transepithelial current flow; *n* = 14 human donors. Panels **E**–**H** are as described for panels **A**–**D**. Data points connected by a line represent paired experiments from a single human donor, graph depicts mean ± standard deviation, and *P* values obtained from paired, 2-sided Student’s *t* tests are presented within the figure.

**Figure 5:**
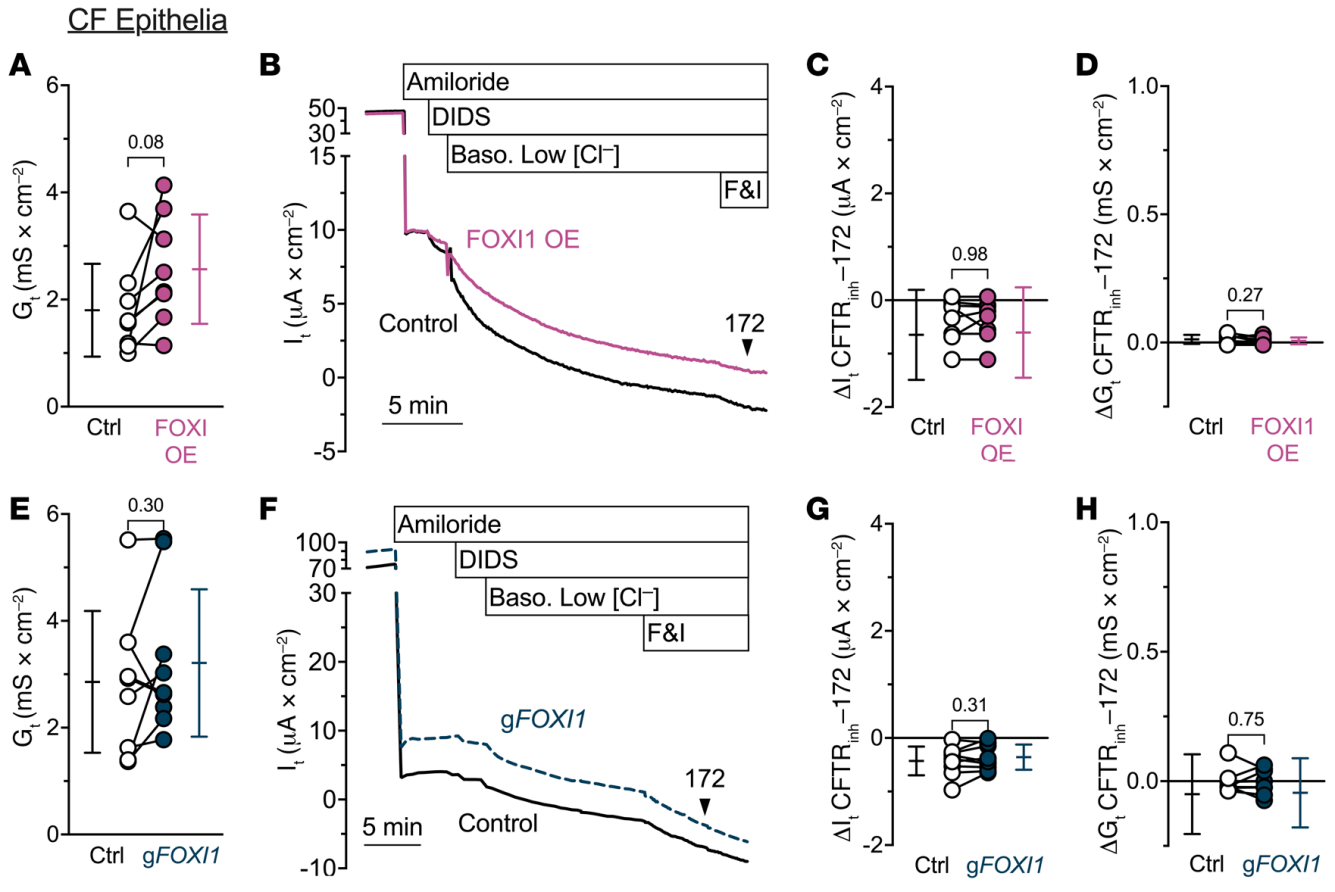
FOXI1 expression in CF epithelia does not alter transepithelial current flow. (**A**–**D**) Increasing ionocyte abundance in CF epithelia does not alter transepithelial current flow; *n* = 8 human CF donors. (**A**) Transepithelial conductances (*G_t_*) obtained from epithelia bathed in symmetrical solutions. (**B**) Example transepithelial current (*I_t_*) recordings with the transepithelial voltage held at 0 mV. The basolateral [Cl^–^] was reduced to drive passive Cl^–^ current. F&I, forskolin and 3-isobutyl-1-methylxanthine. (**C**) CFTR_inh_-172–sensitive *I_t_* data. (**D**) CFTR_inh_-172–sensitive *G_t_* data. (**E**–**H**) Decreasing ionocyte abundance in CF epithelia does not alter transepithelial current flow; *n* = 9 human CF donors. Panels **E**–**H** are as described for panels **A**–**D**. Data points connected by a line represent paired experiments from a single human donor, graph depicts mean ± standard deviation, and *P* values obtained from paired, 2-sided Student’s *t* tests are presented within the figure.

FOXI1 overexpression markedly increased the transepithelial electrical conductance (*G_t_*), whereas decreasing FOXI1 reduced *G_t_* (Figure 4, A and E). These data suggested that the route for Cl^–^ flow through ionocytes is electrically conductive. In additional transepithelial voltage-clamp experiments (*V_clamp_* = 0 mV), we inhibited ENaC with apical amiloride and non-CFTR Cl^–^ channels with apical 4,4′-dilsothiocyano-2,2′-stilbenedifulonic acid (DIDS), and then lowered the basolateral Cl^–^ concentration to provide a chemical driving force, added forskolin and 3-isobutyl-1-methylxanthine (IBMX) to stimulate CFTR, and measured *G_t_* and transepithelial current (*I_t_*) (Figure 4B). The CFTR inhibitor CFTR_inh_-172 blocked much more *I_t_* and *G_t_* in epithelia overexpressing FOXI1 than in control epithelia (Figure 4, C and D). We obtained the opposite results when we performed the same experiments in epithelia with *FOXI1* gene disruption (Figure 4, F–H). Moreover, in CF epithelia lacking CFTR function, neither overexpressing FOXI1 nor disrupting the *FOXI1* gene altered basal *G_t_* (Figure 5, A and E) or the CFTR_inh_-172–dependent changes in *I_t_* and *G_t_* (Figure 5, B–D and F–H). These results indicate that ionocytes provide a CFTR-dependent route for Cl^–^-gradient-driven current flow across the epithelium. This pathway was electrically conductive, suggesting that in addition to apical CFTR, the pathway likely involved an ion channel in the ionocyte basolateral membrane.

### Correlation of ionocyte abundance with liquid absorption and ion flow.

**Figure 6:**
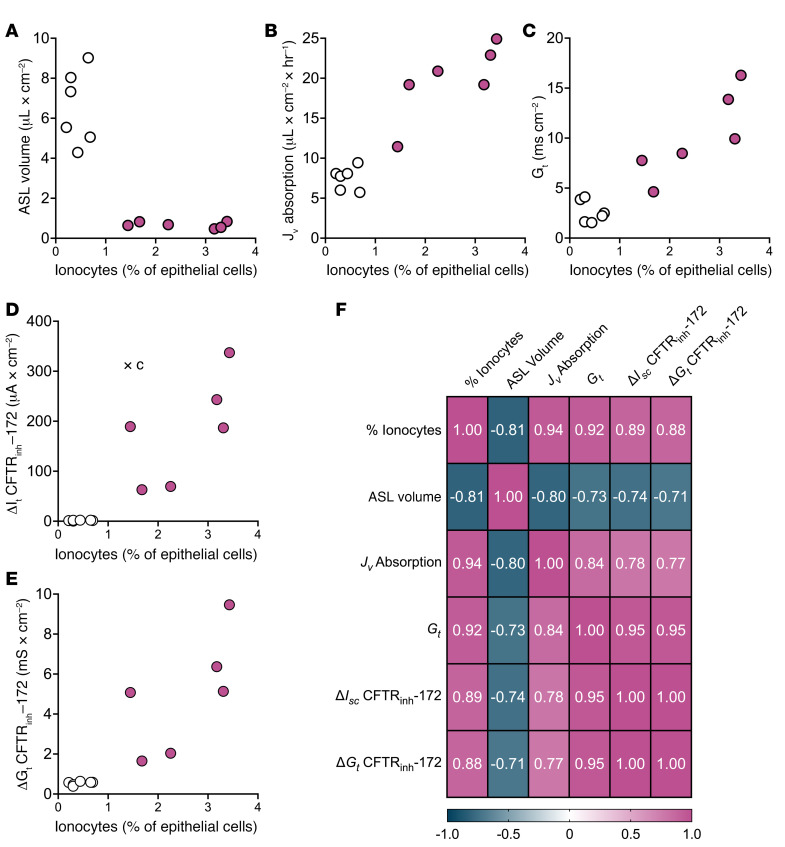
Ionocyte expression correlates with assays of liquid absorption and ion flow. Epithelia were transduced with a lentiviral vector expressing either GFP (open circles) or a bicistronic construct expressing FOXI1 and GFP to generate and label ionocytes (magenta); *n* = 6 human donors. The donors shown were chosen because all experiments were performed within 3 days to provide an accurate estimate of ionocyte abundance. Experiments include (**A**) liquid absorption, (**B**) ASL volume measurements, (**C**) transepithelial conductances (*G_t_*) obtained from epithelia bathed in symmetrical solutions, (**D**) CFTR_inh_-172–sensitive *I_t_* data, and (**E**) CFTR_inh_-172–sensitive *G_t_* data. (**F**) Correlation matrix of Pearson’s *r* values among all variables. *P* < 0.009 for all Pearson’s *r* values.

To assess relationships among ionocyte abundance, transepithelial absorption, and ion flow, we analyzed data from donors on which we performed all assays with and without FOXI1 overexpression. We observed positive correlations among ionocyte abundance, liquid absorption rate, *G_t_*, CFTR_inh_-172–sensitive *I_t_* with a Cl^–^ gradient, and CFTR_inh_-172–sensitive *G_t_*. In addition, all these variables negatively correlated with ASL volume (Figure 6).

### NKCC1 does not affect the rate of liquid absorption.

Ionocytes also contain *SLC12A2* transcripts that encode the Na-K-2Cl cotransporter NKCC1 (1–7). NKCC1 is a basolateral transporter that can carry Cl^–^ into the cell from the basolateral solution. Immunostaining revealed NKCC1 within the same basolateral membrane as barttin, indicating that ionocytes express NKCC1 protein (Supplemental Figure 2A). Therefore, we considered that NKCC1 might partially attenuate the liquid absorption rate. However, inhibiting NKCC1 with basolateral bumetanide did not change liquid absorption in FOXI1-overexpressing epithelia (Supplemental Figure 2B). These data suggest a minimal role for NKCC1 during liquid absorption.

### Ionocytes require basolateral barttin/ClC-K channels to perform liquid absorption.

In considering what channel might provide a pathway for current flow across the basolateral membrane, we were drawn to the possibility that it might be barttin/ClC-K. Serendipitously, barttin is the protein used to identify ionocytes in Figure 1, Figure 2, and by others (14, 15), and barttin, ClC-Ka, and ClC-Kb mRNAs are present in ionocytes but not other airway epithelial cells (4–10). Barttin is a required subunit of human ClC-K Cl^–^ channels (17–19). Further, barttin is a basolateral membrane protein (14, 17) and resides in the basolateral membrane of pulmonary ionocytes (Figures 1C and 2C). Therefore, we hypothesized that barttin/ClC-K Cl^–^ channels are the basolateral ion channel for Cl^–^ absorption.

**Figure 7:**
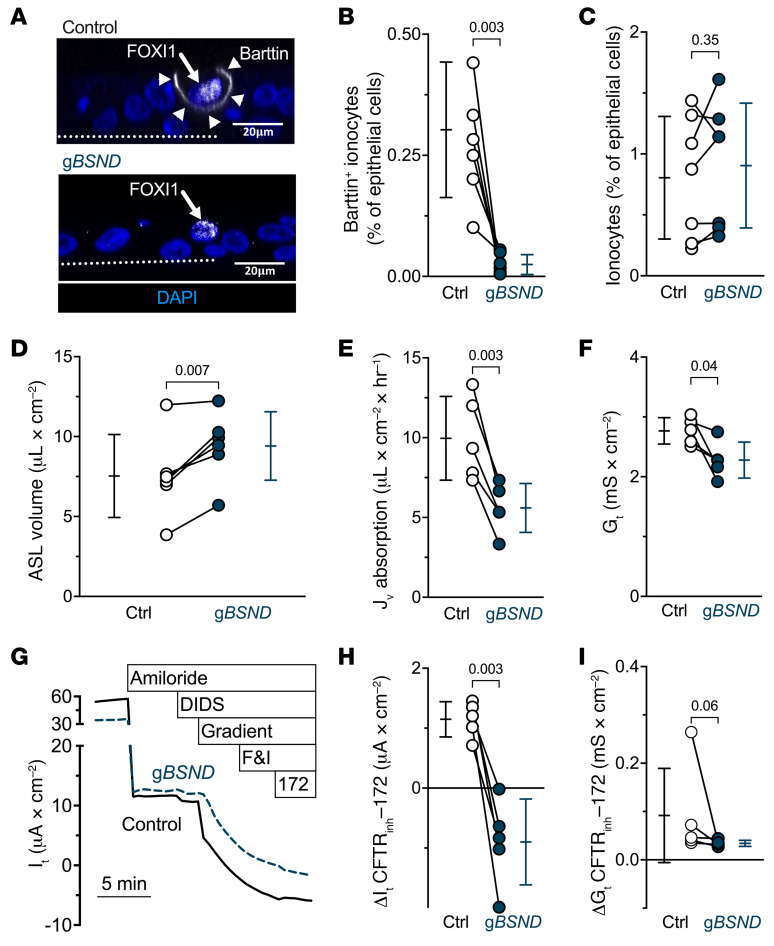
Ionocyte-specific barttin/Cl^–^ channels contribute to liquid absorption and transepithelial current flow. Epithelia were electroporated with CRISPR/Cas9 and guide RNAs (gRNAs) targeting *BSND* (g*BSND*) to decrease the amount of ionocytes that express barttin. (**A**) Confocal images of human airway epithelia. Epithelia were electroporated with CRISPR/Cas9 and g*BSND*. This image pair shows FOXI1 (white in nucleus, white arrow), barttin (white in membrane, white arrowheads), and nuclei (blue). With *BSND*-targeted gene disruption, FOXI1-expressing cells lacked basolateral barttin. Dotted line represents the location of the culture filter. Scale bars: 20 μm. (**B**) Flow cytometric analysis of barttin^+^ epithelial cells for g*BSND* and control epithelia; *n* = 7 human donors. (**C**) Flow cytometric analysis of epithelial cells with the signature NGFR^+^p63^–^ cells, which identifies ionocytes, for g*BSND* and control epithelia; *n* = 7 human donors. (**D**–**I**) Data for control and g*BSND* epithelia (**D**) ASL volume; *n* = 6 human donors. (**E**) Liquid absorption rates; *n* = 5 human donors. (**F**) Transepithelial conductance (*G_t_*) measurements obtained from epithelia bathed in symmetrical solutions. (**G**) Example transepithelial current (*I_t_*) recordings with the transepithelial voltage held at 0 mV. The basolateral [Cl^–^] was reduced to drive passive Cl^–^ current. F&I, forskolin and 3-isobutyl-1-methylxanthine. (**H**) CFTR_inh_-172–sensitive *I_t_* data. (**I**) CFTR_inh_-172–sensitive *G_t_* data. Electrophysiology data include *n* = 5 human donors. Data points connected by a line represent paired experiments from a single human donor, graph depicts mean ± standard deviation, and *P* values obtained from paired, 2-sided Student’s *t* tests — except panel **I**, which was obtained from a 2-sided Wilcoxon’s test — are presented within the figure.

Disrupting the *BSND* gene, which encodes barttin, reduced barttin^+^ cells but left the abundance of ionocytes — indicated by the ionocyte protein expression signature of NGFR^+^p63^–^ cells (2) — unchanged (Figure 7, A–C, and Supplemental Figure 3). With reduced barttin levels, ASL volume increased, and liquid absorption decreased (Figure 7, D and E). In addition, basal values of *G_t_* fell (Figure 7F). Moreover, eliminating a basolateral Cl^–^ conductive pathway attenuated the CFTR_inh_-172–induced changes in *I_t_* and *G_t_* (Figure 7, G–I). These results indicate that ionocytes require barttin/Cl^–^ channels for apical-to-basolateral Cl^–^ flux and ASL absorption.

**Figure 8:**
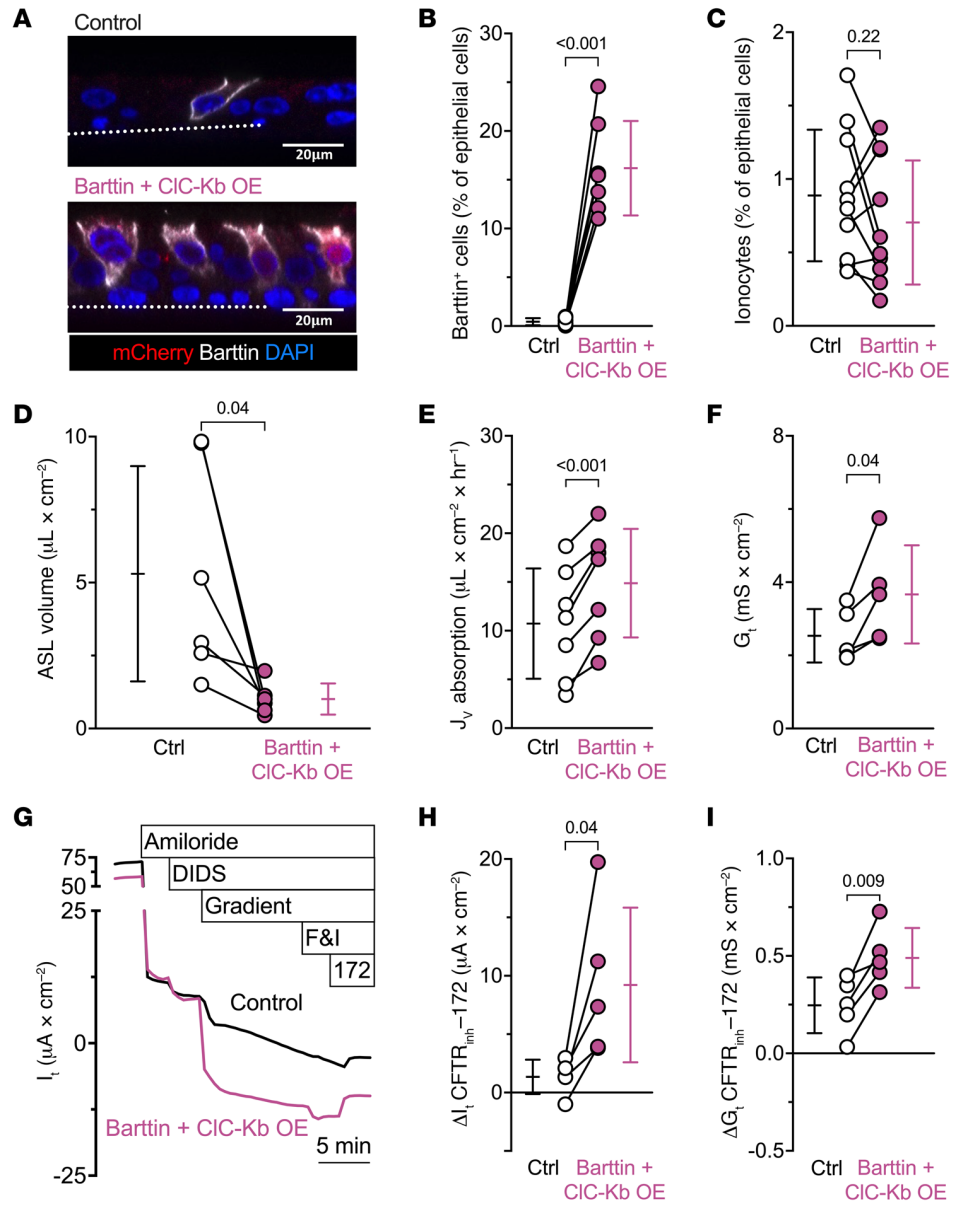
Overexpressing barttin/Cl^–^ channels increases liquid absorption and transepithelial current flow. Epithelia were transduced with an adenoviral vector expressing either GFP or a vector encoding barttin, ClC-Kb, and mCherry (barttin + ClC-Kb OE) to add basolateral Cl^–^ channels to non-ionocyte airway cells. (**A**) The image pair shows mCherry (red), barttin (white), and nuclei (blue). Dotted line represents the location of the culture filter. Scale bars: 20 μm. (**B**) Flow cytometric analysis of barttin^+^ epithelial cells for barttin + ClC-Kb OE and control epithelia; *n* = 7 human donors. (**C**) Flow cytometric analysis of epithelial cells with the signature NGFR^+^p63^–^ cells, which identifies ionocytes, for barttin + ClC-Kb OE and control epithelia; *n* = 10 human donors. (**D**) ASL volume; *n* = 6 human donors. (**E**) Liquid absorption rates; *n* = 7 human donors. (**F**) Transepithelial conductance (*G_t_*) measurements obtained from epithelia bathed in symmetrical solutions. (**G**) Example transepithelial current (*I_t_*) recordings with the transepithelial voltage held at 0 mV. The basolateral [Cl^–^] was reduced to drive passive Cl^–^ current. F&I, forskolin and 3-isobutyl-1-methylxanthine. (**H**) CFTR_inh_-172–sensitive *I_t_* data. (**I**) CFTR_inh_-172–sensitive *G_t_* data. Electrophysiology data include *n* = 5 human donors. Data points connected by a line represent paired experiments from a single human donor, graph depicts mean ± standard deviation, and *P* values obtained from paired, 2-sided Student’s *t* tests are presented within the figure.

We also overexpressed barttin and ClC-Kb from ubiquitous promoters within a single adenoviral vector that would add basolateral Cl^–^ channels to non-ionocyte airway cells. The number of barttin^+^ cells increased without altering ionocyte abundance (NGFR^+^p63^–^ cells) (Figure 8, A–C, and Supplemental Figure 4). Barttin/ClC-Kb overexpression decreased ASL volumes and increased transepithelial liquid absorption rates (Figure 8, D and E), resembling the changes induced by increasing the number of ionocytes (Figure 1, D and F).

Barttin/ClC-Kb overexpression also increased *G_t_* (Figure 8F). Importantly, in epithelia overexpressing barttin/ClC-Kb, CFTR_inh_-172 induced greater changes in *I_t_* and *G_t_* (Figure 8, G–I). These results mirror the changes induced by increasing ionocyte abundance (Figure 4, A–D). They also indicate that CFTR anion channels and barttin/ClC-K anion channels lie in series, thereby providing a pathway for apical-to-basolateral Cl^–^ flow.

## Discussion

**Figure 9:**
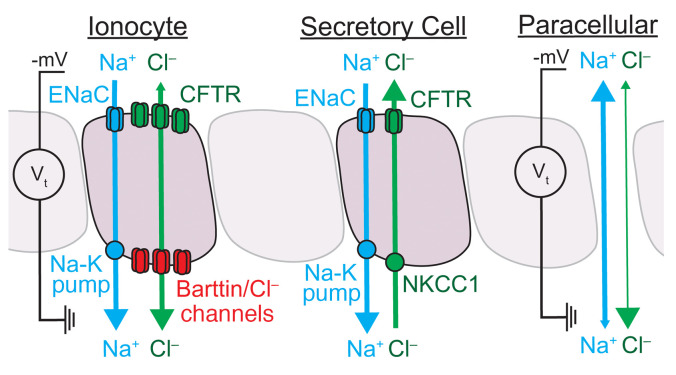
At least 1 paracellular and 2 transcellular pathways exist for Cl^–^ movement across airway epithelia. The model depicts physiological Na^+^ and Cl^–^ flow. The locations of each transporter and channel are based on single-cell RNA sequencing, immunolocalization, and functional data. Ionocyte: The apical CFTR channels (green) and basolateral barttin/Cl^–^ channels (red) permit Cl^–^ movement in either direction in response to the transepithelial electrochemical gradient (green bidirectional arrow). At physiological [Cl^–^], the electrical gradient (*V_t_*) across airway epithelium — primarily established by ENaC (blue in apical membrane) and the Na-K pump (blue in basolateral membrane) — drives Cl^–^ absorption through the ionocyte (depicted by the asymmetry of the green arrowheads). Secretory cell: Secretory cells import Cl^–^ through basolateral NKCC1 (green transporter) to maintain the intracellular Cl^–^ at a concentration that drives Cl^–^ secretion (green unidirectional arrow) through apical CFTR channels (green). The secretory cells also contain a pathway for Na^+^ absorption (blue unidirectional arrow) through ENaC (blue in apical membrane) and the Na-K pump (blue in basolateral membrane). Paracellular pathway: The paracellular region around epithelial cells, depicted as an exaggerated opening between epithelial cells, provides a pathway for ion movement driven by the transepithelial electrochemical gradient. The thicker line for Na^+^ (blue bidirectional arrow) compared with Cl^–^ (green bidirectional arrow) represents the cation selectivity of the paracellular pathway and the dominant arrowhead shows the direction of physiological flow. The influences of ciliated cells and other epithelial cell types on transepithelial Cl^–^ flow remain less characterized and are omitted in the model.

It has long seemed puzzling how airway epithelia manage both ENaC-dependent liquid absorption and CFTR-dependent liquid secretion. Our findings indicate that the airway epithelium accomplishes this by segregating electrolyte absorption and secretion to different cell types (Figure 9). Ionocytes provide a pathway for passive Cl^–^ absorption through apical CFTR channels and basolateral barttin/Cl^–^ channels. The major driving force for Cl^–^ absorption is the negative luminal voltage generated by Na^+^ absorption. Secretory cells provide a pathway for active Cl^–^ secretion through apical CFTR channels (5). Supporting this idea, secretory cells lack barttin/ClC-K channels (1–7), and when we ubiquitously expressed barttin/ClC-Kb channels absorption increased. A Cl^–^ pathway through ionocytes also overcomes the limit on NaCl absorption imposed by a cation-selective — rather than anion-selective — paracellular pathway (14, 15). The ion permeability of the paracellular pathway predicts that most actively absorbed Na^+^ would reflux back into the ASL in the absence of ionocytes. We speculate that the slow liquid absorption rates observed for airway epithelia with *FOXI1* or *BSND* disrupted estimate the capacity of the paracellular pathway to support Na^+^ absorption. Of note, frog and toad skin have some similarity to airway epithelia (1, 2, 16); their abundant principal cells absorb Na^+^, and rare mitochondria-rich cells provide a pathway for Cl^–^ to follow, moving through CFTR channels at the apical membrane and an unidentified anion channel at the basolateral membrane (17–23).

Earlier work revealed a basolateral Cl^–^ conductance in airway epithelia (24–27). Our results suggest that barttin/Cl^–^ channels are responsible. The Cl^–^ channel subunits are likely ClC-Ka and/or ClC-Kb because ionocytes specifically express mRNA transcripts encoding these Cl^–^ channels (1–7). However, we cannot exclude the possibility that an uncharacterized barttin-regulated Cl^–^ channel participates.

Several other groups (1, 2, 28) have also overexpressed FOXI1 and observed an increase in ionocyte mRNAs, similar to the data summarized in Supplemental Table 1, including *BSND* and *CFTR*. In this study, we used a FOXI1-encoding vector at an MOI that produced epithelia with ionocytes comprising 5%–10% of cells and found relationships between ionocytes and measurements that included liquid absorption rate, ASL volume, *G_t_*, CFTR_inh_-172–sensitive *I_t_* with a Cl^–^ gradient, and CFTR_inh_-172–sensitive *G_t_*.

Previous studies of non-CF airway epithelia reported what have seemed to be conflicting observations about how stimulating CFTR by increasing cAMP affects liquid transport. In the presence of apical amiloride, increasing cAMP consistently increased liquid secretion (13, 29, 30). Yet, in the absence of amiloride, mean data show that increasing cAMP increased liquid absorption (13, 29–31). However, the response was variable with different tissue donors, with cAMP increasing absorption in some epithelia and increasing secretion in others. Our findings may provide, at least in part, an explanation. By decreasing transepithelial voltage, amiloride will prevent ionocyte-mediated Cl^–^ and liquid absorption despite increased CFTR activity. Thus, cAMP will stimulate Cl^–^ and liquid secretion via secretory cells. However, in the absence of amiloride, the response to cAMP may be variable; in epithelia with abundant ionocytes, cAMP may induce liquid absorption, and in epithelia with few or no ionocytes, cAMP may induce liquid secretion. Indeed, the number of ionocytes varies widely across epithelia as shown in Figure 1, Figure 2, and Figure 7. Moreover, a study of the porcine submucosal gland duct reported that cAMP increased Cl^–^ absorption in wild-type but not CF pigs (32). We speculate that ionocytes in this epithelium (33) may explain absorption induced by cAMP. CFTR within the acinus and ducts of the airway epithelium could be analogous in function to the sweat gland, where β adrenergic stimulation triggers Cl^–^ secretion through CFTR in the secretory coil and Cl^–^ absorption through CFTR in the duct.

It is interesting that in addition to basolateral barttin/Cl^–^ channels, ionocytes also express basolateral NKCC1. The primary function of NKCC1 in ionocytes might be to regulate cell volume and K^+^ secretion. However, ionocytes can secrete Cl^–^ when the transepithelial voltage is held at 0 mV with the short-circuit current technique in conditions with symmetrical solutions and the ENaC inhibitor amiloride (1, 2, 28). This condition shifts the electrochemical gradient to favor Cl^–^ secretion. Therefore, it remains possible that ionocytes could secrete Cl^–^ under certain conditions, perhaps in response to extracellular and intracellular regulators. Much remains to be learned about how the individual transport processes in ionocytes are regulated. The recent report that the phosphodiesterase PDE1C dampens the effect of forskolin in FOXI1-overexpressing epithelia provides one potential regulator (28).

Our findings may also have implications for CF airway disease. The observation that CFTR is critical for Cl^–^ absorption, in addition to Cl^–^ secretion, indicates that both processes are disrupted in CF. Thus, how loss of CFTR function influences the amount of ASL may depend, in part, on the relative abundance of ionocytes versus secretory cells. The relative contributions of other ion channels or residual CFTR function in ionocytes could also influence ASL volume.

The discovery of ionocytes raised the question of whether CF gene therapies should target this cell type (34). Restoring CFTR in secretory cells may be key for CF gene therapy because people with Bartter syndrome — caused by mutations in barttin or ClC-K Cl^–^ channels — do not develop CF airway disease. Targeting corrective genetic therapies to airway progenitor cell types, such as basal cells, should ensure that both progeny secretory cells and ionocytes will perform their physiological functions.

## Methods

### Lentiviral expression vectors.

The cDNA for human *FOXI1* (gene ID: 2299) was obtained from the DNASU Plasmid Repository (https://dnasu.org/DNASU/Home.do) (clone ID: HsCD00813494) and cloned into the pSSIN/SFFV-IRES-GFP vector (a gift from Anusha Sridharan, Cincinnati Children’s Hospital Medical Center, Cincinnati, Ohio, USA) by in-fusion cloning (Takara Bio). The resultant pSSIN/SFFV-hFOXI1-IRES-GFP plasmid was used for HIV lentivirus vector production at the University of Iowa Viral Vector Core (https://medicine.uiowa.edu/vectorcore/). The lentivirus was pseudotyped with the VSV-G envelope. Viral titers were determined by flow cytometry (TU/mL) and digital drop PCR assay (IGU/mL).

### Culturing primary human airway epithelial cells and lentivirus transduction.

The protocol for obtaining and culturing human airway epithelial cells was approved by the University of Iowa Institutional Review Board. Airway epithelial cells from non-CF donors were isolated from trachea or bronchi from postmortem lungs deemed not suitable for transplant, whereas CF tissues were obtained after lung transplant. The genotypes of the CF donors were c.[1521_1523delCTT];[1521_1523delCTT] (ΔF508/ΔF508, *n* = 7) and c.[1521_1523delCTT ];[1585-1G>A] (Δ508/1717-1G->A, *n* = 2). Cell suspensions were prepared in USG culture medium, a 1:1 mixture of DMEM/F12 supplemented with 2% Ultroser G (Sartorius Stedim). The cell suspension was transduced with an HIV-based lentivirus (MOI = 4) and hexadimethrine bromide (Polybrene) at a final concentration of 2 μg/mL. A VSV-G lentivirus expressing GFP served as a control. The cell-virus mixture was then seeded onto collagen-coated, semipermeable membranes (0.33 cm^2^, no. 3413 polycarbonate, Corning Costar Transwell Permeable Supports) and differentiated at the ALI as previously described (35). Epithelial cells were studied at least 21 days after seeding. Initial transepithelial conductance (*G_t_*) values are reported throughout this paper and corresponding transepithelial resistance (*R_t_*) measurements are available in the Supporting Data Values XLS file.

### Genetic disruption of FOXI1 and BSND genes in human airway epithelia.

The guide RNA was designed using the online software Benchling (https://www.benchling.com/) and synthesized by Integrated DNA Technologies (IDT). For each target gene, exon sequences from the GRCh38 (hg38, *Homo* *sapiens*) genome were used to design 20-nucleotide-long guide RNAs with a 3′ NGG protospacer adjacent motif (PAM). All guide RNAs selected had a Benchling on-target score of greater than 60 and an off-target score of greater than 50 (36–37). The online program inDelphi (https://indelphi.giffordlab.mit.edu/gene) was used to predict the frameshift frequency and further assess the on-target efficiency of the selected guide RNA sequences (38). Parameters used for inDelphi analysis included the human genome (hg38), the *FOXI1* or *BSND* gene, and the HEK293 cell type. Guide RNA sequences with “high” or “very high” precision and frameshift frequency scores were then synthesized as *crispr* (crRNA) by IDT. Specific information for the guides ultimately used for experiments are in Supplemental Tables 2 and 3.

Guide RNAs were created by combining crRNA with tracrRNA (IDT, 1072532) at equimolar concentrations (100 μM), annealing them by incubating the mixture at 95°C for 10 minutes, and renaturing them to a single guide RNA (sgRNA) by returning the mixture to room temperature. Ribonucleoprotein (RNP) was prepared by combining the sgRNA and Alt-R SpCas9 Nuclease V3 (IDT, 1081058) in Dulbecco’s PBS (Gibco). To deliver guide RNA and Cas9 to human airway epithelial cells, cells in suspension from a human donor were seeded onto a collagen-coated 6-well plate and cultured in PneumaCult Ex-Plus culture medium (Stemcell Technologies). When cells reached 80% confluence, they were dissociated with TrypLE Express Enzyme (Gibco Laboratories), pelleted by 120*g* centrifugation for 10 minutes, and resuspended in PneumaCult Ex-Plus culture medium. Each 100 μL nucleofection reaction consisted of nucleofection buffer solution (Amaxa Basic Nucleofector Kit for Primary Mammalian Epithelial Cells, Lonza), 1 × 10^6^ cells, 1 μL Cas9 electrophoretic enhancer (IDT, 1075915), and 7.5 μL of each RNP used. The cell suspension was electroporated with Nucleofector 2b (Lonza, AAB-1001) using its U-024 program. After electroporation, cells were seeded onto a collagen-coated 6-well plate with prewarmed PneumaCult Ex-Plus culture media and incubated at 37°C and 5% CO_2_ overnight. The next day, unattached cells were washed away with PBS, and culturing in PneumaCult Ex-Plus culture medium resumed. When the cells reached 80% confluence, they were dissociated with TrypLE and 1.2 × 10^5^ cells were seeded onto 0.33 cm^2^ semipermeable membranes submerged in PneumaCult Ex-Plus. After 2 days, the apical media were removed, and the basolateral culture media were changed from PneumaCult Ex-Plus to PneumaCult ALI. Cells were cultured at the ALI more than 3 weeks prior to experiments.

Genomic DNA was extracted using QuickExtract (Lucigen), and a PCR with genomic DNA was performed using a KAPA HiFi Readmix kit (Roche) and primers designed to bind regions outside of the targeted sequence (Supplemental Figures 5 and 6). PCR products were confirmed by sequencing.

### Adenovirus 5 expression vectors.

The cmv-*bsnd*-pause-sffv-*clcnkb*-T2A-mCherry plasmid carrying the coding sequences for human barttin (barttin CLCNK type accessory subunit β; gene ID: 7809) and ClC-Kb (chloride voltage-gated channel Kb; gene ID:1188) in 2 open reading frames was synthesized by GeneScript and inserted into an adenovirus 5 (Ad5) vector at the E1 position. The resultant Ad5-cmv-*bsnd*-pause-sffv-*clcnkb*-T2A-mCherry plasmid was used for Ad5 viral vector production at the University of Iowa Viral Vector Core (https://medicine.uiowa.edu/vectorcore/). Prior to infecting differentiated human airway epithelia, epithelia were apically treated with 6 mM EGTA/10 mM HEPES to disrupt tight junctions. After 2 hours, the apical EGTA/HEPES solution was aspirated and cells were apically transduced with Ad5-CLCNKB/BSND (MOI = 30). After an additional 4 hours, the apical liquid was aspirated, and cultures were studied 5 days later.

### Reverse transcriptase quantitative PCR.

Primers used for reverse transcriptase quantitative (RT-qPCR) were designed and validated using standard procedures. RNA was isolated from primary cultures using TRIzol and the RNeasy kit according to the manufacturer’s instructions (Qiagen). cDNA was prepared using the High-Capacity cDNA Reverse Transcription kit (Life Technologies) with random hexamers. Power SYBR PCR master mix (Life Technologies) was used for amplification, and the primers used are listed in Supplemental Tables 2 and 4.

### Immunofluorescent staining and confocal microscopy.

At the time of immunostaining, human airway epithelia were briefly washed 3 times with PBS, fixed with 4% paraformaldehyde for 15 minutes, briefly washed 3 times with PBS, permeabilized with 0.2% Triton X-100 for 20 minutes, briefly washed 3 times with PBS, and then blocked with 10% filtered normal goat serum (Thermo Fisher Scientific) in superblock buffer (Thermo Fisher Scientific) for 1 hour. Cultures were then incubated for 1 hour at room temperature with the primary antibody diluted in blocking buffer. Antibodies used in this study include monoclonal mouse anti–human CFTR (1:50, catalog 596, CFTR Antibodies Distribution Program, Cystic Fibrosis Foundation), polyclonal rabbit anti–human FOXI1 (1:300, catalog ab153935, Abcam), polyclonal rabbit anti–human barttin (1:300, catalog ab196017, Abcam, sold under its gene name *BSND*). After incubation, epithelia were briefly washed 3 times with PBS and then incubated with secondary antibodies diluted in blocking buffer for 1 hour at room temperature. Secondary antibodies included Alexa Fluor 568–labeled goat anti-mouse (1:500, catalog A-11004, Life Technologies) and Alexa Fluor 647–labeled goat anti-rabbit secondary (1:500, catalog A32733, Life Technologies). The primary and secondary antibodies were applied to both the apical and basolateral surfaces of cultured cells. After briefly washing the epithelia, membranes were separated from the plastic cylinder by cutting the edges with a razor blade and mounted on slides with Vectashield with DAPI (4′,6-diamidino-2- phenylindole; Vector Laboratories, Inc.). For Figure 3A, anti-FOXI1 and -barttin antibodies were from the same species and obtained from the same channel. We could differentiate between signals because FOXI1 is nuclear, whereas barttin resides in the basolateral membranes. Fluorescence images were captured using a confocal microscope (Leica Microsystems, Inc.). Images were analyzed using ImageJ (v2.3.0/1.53f, NIH). For CFTR mAb 596, immunostaining included in Figures 1 and 2 show that the signal for CFTR mAb596 is apical on ionocytes of non-CF epithelia and cytosolic in ionocytes of CF epithelia. We did not use this antibody beyond this demonstration. The barttin antibody failed to label epithelia when the *BSND* gene is disrupted (Figure 7B), and the FOXI1 antibody labels the same cells as ionocyte-specific barttin (Figure 7A).

### Flow cytometry.

Differentiated human airway epithelia were incubated with fixable Live/Dead staining (Invitrogen) for 15 minutes before washing twice with PBS. Then, cultures were incubated with Accutase cell dissociation solution (Innovative Cell Technologies, Inc.) for 15 minutes at 37°C. Single-cell suspensions were collected and centrifuged at 250*g* for 10 minutes. GFP^+^ or mCherry^+^ cells could be quantified directly by flow cytometry. For antibody staining, cells were fixed with 4% paraformaldehyde for 30 minutes, and then permeabilized using the eBioscience FOXP3/Transcription factor staining buffer set (Invitrogen). Cells were blocked overnight at 4°C with 10% normal goat serum flow buffer. Antibodies were diluted in 10% flow buffer and incubated for 1 hour at room temperature. Antibodies included PE/Dazzle 594 NGFR/CD271 (catalog 345120, BioLegend, mouse monoclonal antibody; 1:500), P63-488 (catalog NBP3-08736AF488, Novus, rabbit monoclonal antibody; 1:1000), and BSND 568 (catalog Ab196017, Abcam, rabbit polyclonal antibody; 1:1000). After incubation with primary antibodies, cells were washed 3 times with flow buffer and resuspend in 200 μL flow buffer. Data were collected using an Attune NxT flow cytometer (Invitrogen) and analyzed by FlowJo software. Example plots are shown in Supplemental Figures 1–4.

### ASL volume.

ASL volumes of cultured human airway epithelia were estimated using a previously described approach where the meniscus is imaged by its attenuation of transmitted light and volume obtained from a calibration curve (39). Briefly, cultured human airway epithelia were treated overnight with 10 μM forskolin and 100 μM IBMX before imaging, and brightfield images were acquired with a Leica CTR6000 inverted microscope using the 2.5×/0.07 HC FL PLAN objective lens and Leica Application Suite 5 software. For each donor, 2–4 technical replicates were acquired per condition. Acquired intensities were analyzed using ImageJ (v2.3.0/1.53f, NIH) by calculating the area under the curve for four 1-mm line segments drawn from the edge of the filter toward its center on 4 separate sides of the filter. The ASL volume for each *n* was interpolated from a calibration curve using the mean technical replicate value, which included 2 to 4 condition replicates and 4 line segments for each replicate. The calibration curve was created by aspirating the ASL, and then adding normal saline of known volumes to the epithelial surface.

### Liquid absorption (J_v_) assay.

Liquid absorption was measured using methods previously described (29). Briefly, cultured epithelia were treated overnight with 10 μM forskolin and 100 μM IBMX. Epithelia were washed with PBS, and then assayed with 10 μM forskolin and 100 μM IBMX to maximally activate CFTR. We applied 60 μL of saline buffer to the apical surface containing (mM): 137.8 NaCl, 4 KCl, 29 NaHCO_3_, 1.2 CaCl_2_, 0.6 MgCl_2_, and 1 NaH_2_PO_4_. After a 4-hour incubation at 37°C/5% CO_2_, the apical solutions were collected under mineral oil and their volume was measured with microcapillary tubes (Drummond). Some experiments contained 100 μM amiloride in the apical solution or 100 μM bumetanide in the basolateral solution during the 4-hour assay period.

### Ussing chamber assays.

Cultured epithelial cells were mounted in Ussing chambers (Physiologic Instruments, Inc.) and initially bathed in symmetrical Cl^–^ buffered solutions. Symmetrical Cl^–^ solutions consisted of (mM): 135 NaCl, 5 HEPES, 0.6 KH_2_PO_4_, 2.4 K_2_HPO_4_, 1.2 MgCl_2_, 1.2 CaCl_2_, 5 dextrose, pH titrated to 7.40 at 37°C with NaOH. Low-Cl^–^ solutions consisted of (mM): 135 Na gluconate, 5 HEPES, 0.6 KH_2_PO_4_, 2.4 K_2_HPO_4_, 1.2 MgCl_2_, 1.2 CaCl_2_, 5 dextrose, pH titrated to 7.40 at 37°C with NaOH. The command voltage was set to 0 mV and short-circuit current (*I_sc_*) was monitored. A periodic ±5 mV pulse was applied across the epithelium to compute transepithelial conductance (*G_t_*). The following drugs were used in the Ussing chambers: apical amiloride (Sigma-Aldrich, 100 μM from 100 mM DMSO stock) to inhibit ENaC, apical DIDS (Sigma-Aldrich, 100 μM from 100 mM DMSO stock) to inhibit non-CFTR Cl^–^ channels of airway epithelia (40), forskolin (Cayman Chemical, 10 μM from 10 mM DMSO stock), IBMX (Sigma-Aldrich, 100 μM from 100 mM ethanol stock), and CFTR_inh_-172 (Sigma-Aldrich, 10 μM from 10 mM DMSO stock). Electrophysiological assays were performed in parallel to other assays.

### Statistics.

Prism (GraphPad Software) was used to perform all statistical tests. For each data set, normality was tested using the D’Agostino-Pearson normality test. Depending on normality, either a paired 2-sided Student’s *t* test, a 2-sided Wilcoxon’s matched-pairs signed-rank test, or a repeated-measure 1-way ANOVA was performed. All tests and sample sizes are reported in each figure legend and all *P* values are reported within each figure. A *P* value of less than 0.05 was considered significant. All data are presented as mean ± the standard deviation of the mean.

### Study approval.

All studies were approved by the University of Iowa Institutional Review Board.

### Data availability.

The Supporting Data Values XLS file includes values for each data point presented in the paper. Further data supporting the findings of this study are available from the corresponding authors upon reasonable request.

## Author contributions

LL, ST, GSRI, DKM, JZ, DAS, PLS, MJW, IMT, and PBM designed the studies and interpreted data. LL, ST, PHK, TR, and IMT performed experiments. LL, MJW, IMT, and PBM wrote the manuscript. LL, ST, GSRI, PHK, TR, DKM, JZ, DAS, PLS, MJW, IMT, and PBM approved the final version of the manuscript.

## Supplementary Material

Supplemental data

Supporting data values

## Version 1 08/15/2023

In-Press Preview

## Version 2 10/16/2023

Electronic publication
