# Supplementary material for: CFTR-rich ionocytes mediate chloride absorption across airway epithelia
Source: J Clin Invest. 2023 Oct 16;133(20):e171268. doi: 10.1172/JCI171268 (PMC10575720; doi:10.1172/JCI171268)
Supplement: Supplemental data [file jci-133-171268-s205.pdf]

Supplementary Materials

CFTR-rich ionocytes mediate chloride absorption across airway epithelia

Lei Lei[1], Soumba Traore[1], Guillermo S. Romano Ibarra[2], Philip H. Karp[2,3], Tayyab Rehman[2†], David K. Meyerholz[4], Joseph Zabner[2], David A. Stoltz[2,5,6], Patrick L. Sinn[1,7], Michael J. Welsh[2,3,6], Paul B. McCray Jr.,[1,7]* and Ian M. Thornell[2]*

[1]Stead Family Department of Pediatrics and Pappajohn Biomedical Institute Roy J. and Lucille A. Carver College of Medicine, University of Iowa, Iowa City, IA 52242, USA;
[2]Department of Internal Medicine and Pappajohn Biomedical Institute Roy J. and Lucille A. Carver College of Medicine, University of Iowa, Iowa City, USA;
[3]Howard Hughes Medical Institute, University of Iowa, Iowa City, USA;
[4]Department of Pathology, Roy J. and Lucille A. Carver College of Medicine, University of Iowa, Iowa City, USA;
[5]Department of Biomedical Engineering, University of Iowa, Iowa City, USA;
[6]Department of Molecular Physiology and Biophysics, Roy J. and Lucille A. Carver College of Medicine, University of Iowa, Iowa City, USA;
[7]Department of Microbiology and Immunology, Roy J. and Lucille A. Carver College of Medicine, University of Iowa, Iowa City, USA.

[†]Present address: Department of Internal Medicine, University of Michigan, Ann Arbor, USA

*These authors contributed equally to this work.

Address correspondence to:

Ian M. Thornell
169 Newton Road
6332 PBDB
Iowa City, IA 52242 USA
Phone: (319) 335-7574
Email: ian-thornell@uiowa.edu

Paul B. McCray Jr.
169 Newton Road
6320 PBDB
Iowa City, IA 52242 USA
Phone: (319) 356-1828
Email: paul-mccray@uiowa.edu

Conflict of Interest Statement: The authors have declared that no conflict of interest exists.

Supplemental Table 1

**Target**	**Fold-change from control**	***P* value**
FOXI1	441.5 ± 101.4	0.0032
CFTR	7.7 ± 1.7	0.0045
BSND	116.7 ± 30.3	0.0047
CLCNKA	1.3 ±0.6	0.3937
CLCNKB	20.4 ± 5.4	0.0056
NGFR	2.9 ± 1.2	0.0551
ASCL3	43.4 ± 12.5	0.0065
RARRES2	5.5 ± 3.4	0.0763
ATP6V0D2	244.0 ± 38.2	0.0010
ATP6V0B	2.8 ± 2.0	0.1824
ATP6V1B1	4.0 ± 2.0	0.0554
TMPRSS11E	44.9 ± 11.3	0.0044
CD24	2.1 ± 0.3	0.0069
PPARG	1.9 ± 0.6	0.0570
FOXI2	4.0 ± 1.3	0.1012

Supplemental Table 1 RT-qPCR of ionocyte transcripts for FOXI1-OE vs. control epithelia. Each FOXI-OE sample represents the fold-change from its donor-matched control epithelia. n = 4 human donors. Error represents standard deviation of the mean. P values were obtained using the one-sample t test with a hypothetical fold-change of 1.

Supplemental Table 2

**Guide**				
**Target**	**Exon**	**Guide 1**	**Guide 2**	**Deletion Length**
FOXI1	1	CATAGTAGAGGTTCATCTCGGGG	ACAACAAGAGCAAGGCCGGCTGG	412 base pairs
BSND	1	CCAGCACAATGAAGCCGATCCGG	TAGAAGGTGCCGTAGACCTGGGG	70 base pairs
**PCR Primers**				
**Target**		**Forward**	**Reverse**	**Ref ID**
FOXI1		5'-TCTAGCATGTCATTAGTGGGGACCT	5'-TGGAGATGCAGATGAATCGTAAT	NM_012188.5
BSND		5'-TCTCTCCCTGTGTAAGCCTGT	5'-GGGGTCAGCGCTCATTAGCT	NM_057176.3

Supplemental Table 2 Guide RNA and qPCR primers used to evaluate efficiency. The upper portion of the table lists our guide sequences used to disrupt target genes and their predicted base pair deletion length. The lower portion of the table lists our primers pairs used for genomic PCRs.

Supplemental Table 3

**Target**	**Guide**	**Benchling Scores**		**inDelphi Scores**	
		On Target	Off Target	Precision Score (Percentile)	Frameshift Frequency (Percentile)
FOXI1	1	60.9	86.4	93.9	84.3
	2	55.7	68.9	97.8	88.8
BSND	1	48.2	84.0	98.4	75.1
	2	65.9	79.5	79.3	85.3

Supplemental Table 3 Scores for guide RNAs used in this study. The table lists scores for guide sequences first obtained using Benchling then verified using inDelphi. References for the Benchling and inDelphi algorithms are provided within Methods.

Supplemental Table 4

Target	Forward	Reverse	Ref ID
RARRES2	5'-GAGGGACTGGAAGAAACCCGA	5'-CTGTCCAGGGAAGTAGAAGCTGT	NM_002889.4
ASCL3	5'-GGACAACAGAGGCAACTCTAGT	5'-AGGGGCAGGGTTCACTGTAAT	NM_020646.3
ATP6V0D2	5'-TCTGATCGAAACGCCATTAGC	5'-CTTCTTTGCTCAATTCAGTGCC	NM_152565.1
ATP6V0B	5'-CCATCGGAACTACCATGCAGG	5'-TCCACAGAAGAGGTTAGACAGG	NM_004047.5
ATP6V1B1	5'-CAAGGCGATTGTTCAGGTGTT	5'-TCCCCTGTAAATTCGCAAGTG	NM_001692.4
FOXI1	5'-GAACTCCATCCGCCACAACC	5'-GGCTGTGCTAGAGGAAACATCT	NM_012188.5
CLCNKB	5'-GTGGGCATAGTGCGAAGGG	5'-CAAAGAGGTTGTGTGCCTCAT	NM_001165945.2
TMPRSS11E	5'-CAGTGGGATGGGAGTCATCG	5'-CTGGCAGGGTTCTTATATGTTGT	NM_014058.4
CFTR	5'-AGTGGAGGAAAGCCTTTGGAGT	5'-ACAGATCTGAGCCCAACCTCA	NM_000492.4
CLCNKA	5'-ACCCTGACGCTATTCTCAGAG	5'-CGATGTCACGAAGAGGGACTG	NM_004070.4
BSND	5'-GGAAAGACGCCTAACTCAGAGC	5'-CCTCCCTGTCATGTGGAGATG	NM_057176.3
TMEM61	5'-CACCCACGGAGTATCCGGT	5'-CACAGTGAGGTAGTACAGGTCT	NM_182532.3
CD24	5'-CTCCTACCCACGCAGATTTATTC	5'-AGAGTGAGACCACGAAGAGAC	NM_013230.3
NGFR	5'-CCGTTGGATTACACGGTCCAC	5'-TGAAGGCTATGTAGGCCACAA	NM_002507.4
PPARG	5'-ACCAAAGTGCAATCAAAGTGGA	5'-ATGAGGGAGTTGGAAGGCTCT	NM_001354668.2
FOXI2	5'-CTGGCGGGCGACTTTTCTT	5'-GGGAGGGGTTAAGGGTCTG	NM_207426.3

Supplemental Table 4 RT-qPCR primers used in this study.

Supplemental Figure 1

A Figure 1A Example Plots

Control

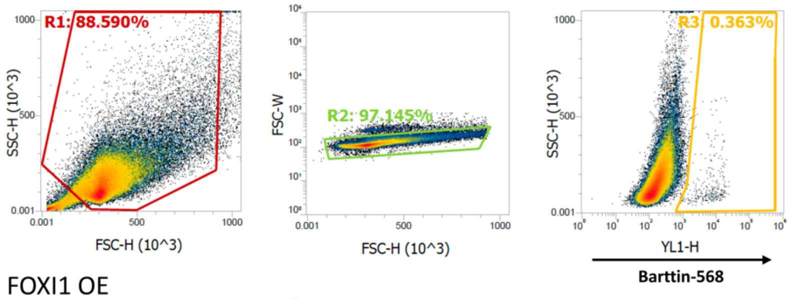

FOXI1 OE

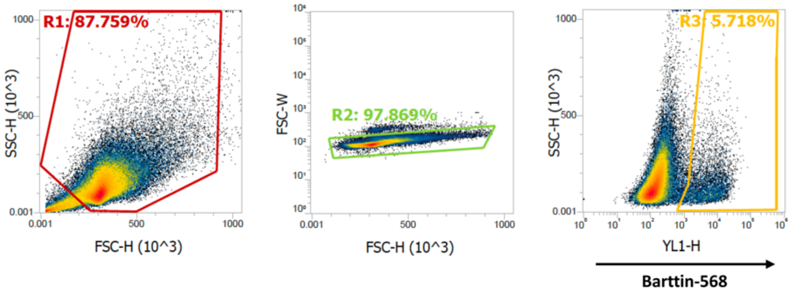

B Figure 1B Example Plots

Control

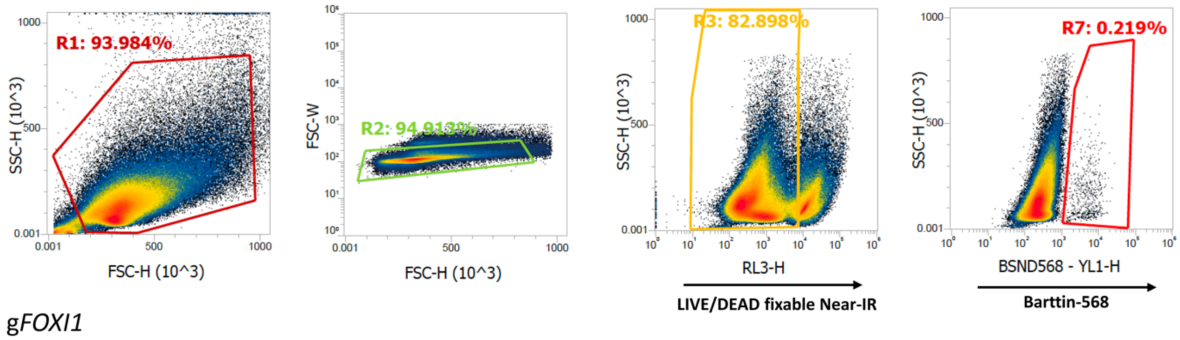

g*FOXI1*

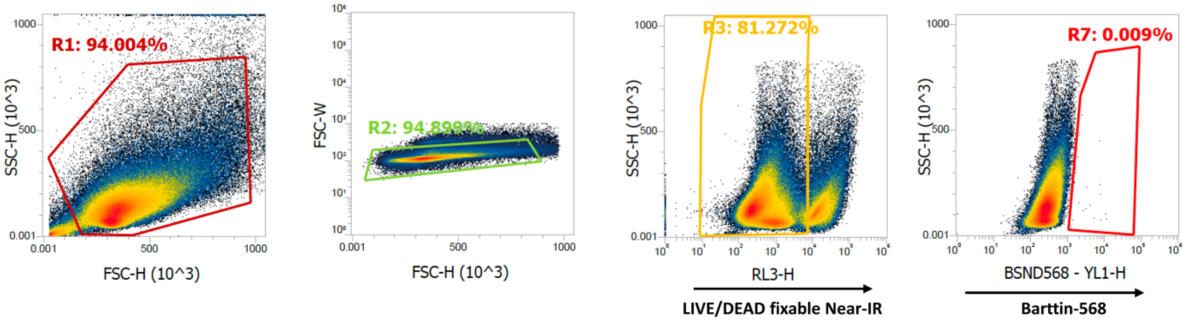

Supplemental Figure 1 Example flow cytometry analysis for Figure 1, A and B. Epithelia were transduced with a lentiviral vector expressing either GFP or a bicistronic construct expressing FOXI1 and GFP to generate and label ionocytes (FOXI1-OE), or they were electroporated with CRISPR/Cas9 and guide RNAs (gRNA) targeting *FOXI1* (g*FOXI1*) to decrease ionocyte abundance. Cells were gated by light intensities detected by a flow cytometer. **(A)** Example **Figure 1A**-type experiments with FOXI-OE epithelia and controls. The gating scheme is shown from left to right. Side scatter height (SSC-H) vs. forward scatter height (FSC-H) was used to select for large and granular cells. FSC-width (FSC-W) vs. FSC-H was used to exclude cellular debris and clumping. Barttin was detected within the selected population. **(B)** Example **Figure 1B**-type experiments for g*FOXI1* epithelia and controls. The first 2 gates are as described for Panel **A**. Then, cells were gated for intact plasma membranes, then barttin. CF epithelia (**Figure 2, A and B**) underwent identical gating.

Supplemental Figure 2

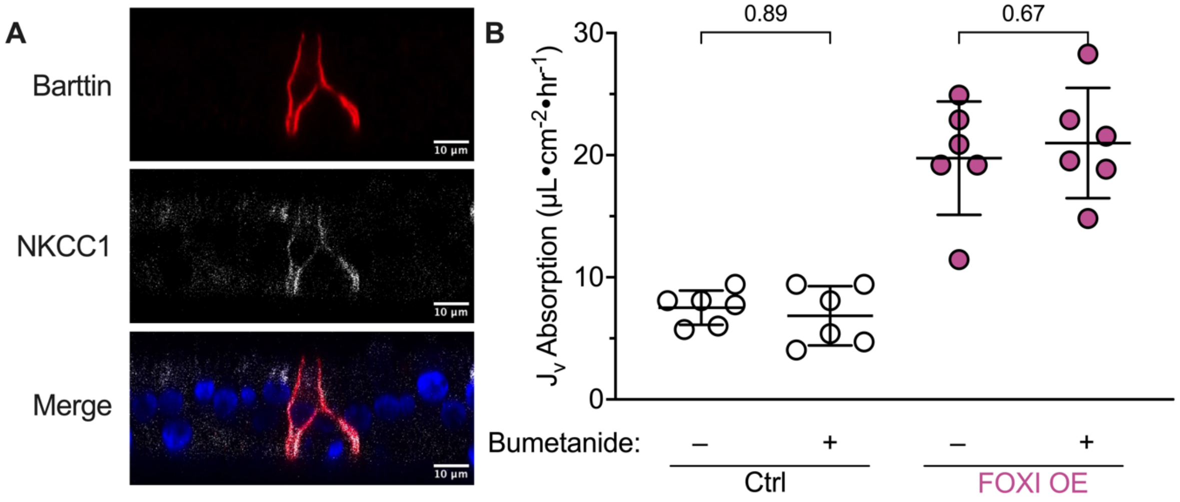

Supplemental Figure 2 NKCC1 does not influence liquid absorption rates. (A) Confocal images of human airway epithelia. The image shows barttin (white), NKCC1 (red), and nuclei (blue); scale bars = 10 µm. **(B)** Epithelia were transduced with a lentiviral vector expressing either GFP or a bicistronic construct expressing FOXI1 and GFP to generate and label ionocytes (FOXI1 OE), then liquid absorption rates were obtained with 100 µM bumetanide or DMSO in the basolateral solution; *n* = 6 human donors. Graph depicts mean ± standard deviation, and Šídák-corrected *P* values obtained from a repeated measure one-way ANOVA are presented within the figure.

Supplemental Figure 3

A Figure 7B Example Plots

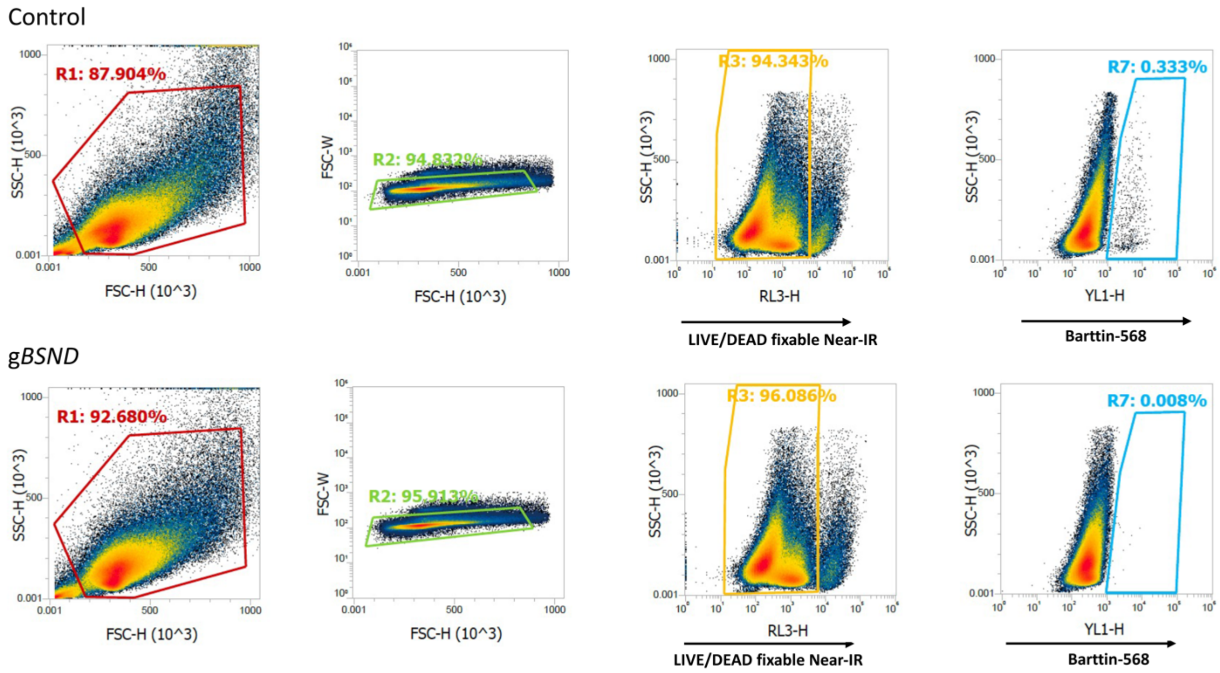

B Figure 7C Example Plots

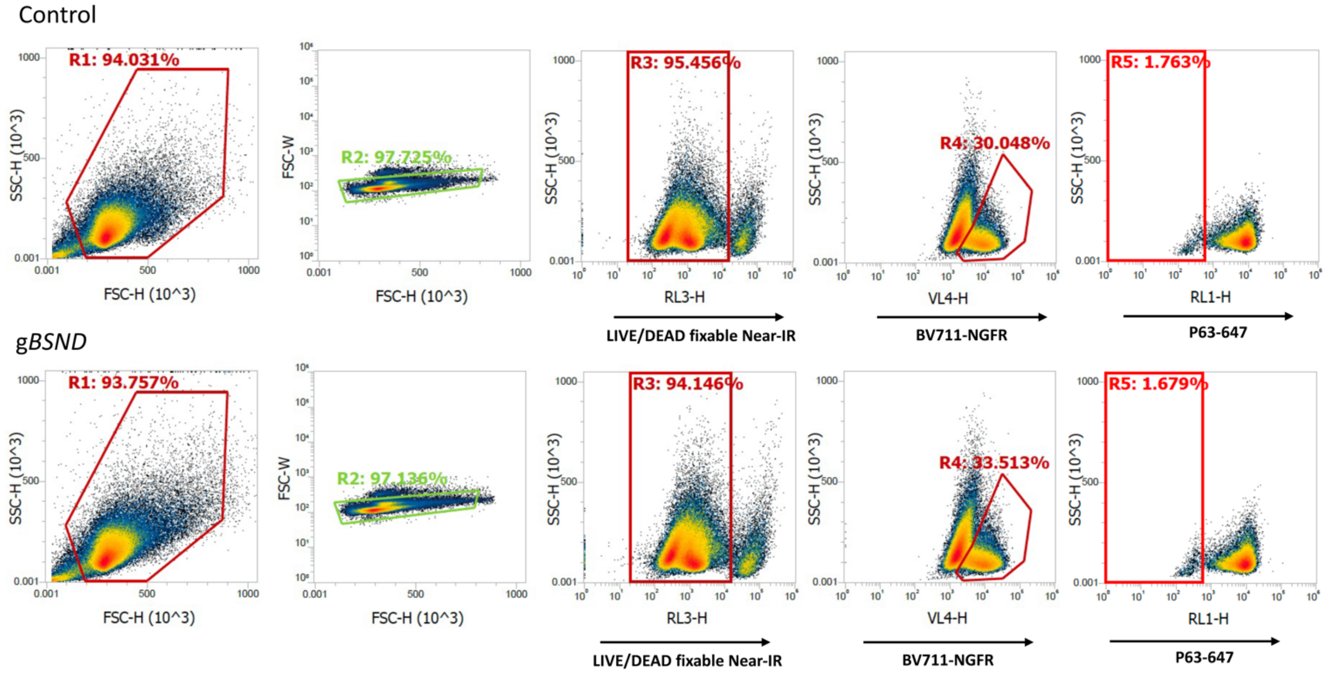

Supplemental Figure 3 Example flow cytometry analysis for Figure 7, B and C. Epithelia were electroporated with CRISPR/Cas9 and guide RNAs (gRNA) targeting *BSND* (g*BSND*) to decrease the amount of ionocytes that express barttin. Cells were gated by light intensities detected by a flow cytometer. **(A)** Example **Figure 7B**-type experiments. The gating scheme is shown from left to right. Side scatter height (SSC-H) vs. forward scatter height (FSC-H) was used to select for large and granular cells. FSC-width (FSC-W) vs. FSC-H was used to exclude cellular debris and clumping. Then, cells were gated for intact plasma membranes, then barttin. **(B)** Example **Figure 7C**-type experiment. The first 3 gates are as described for panel **A**. Additionally, cells were gated for NGFR (neuronal growth factor receptor) expression, which is on basal cells and ionocytes, then p63 was used to separate basal cells ($p63^+$) from ionocytes ($p63^-$).

Supplemental Figure 4

A Figure 8B Example Plots

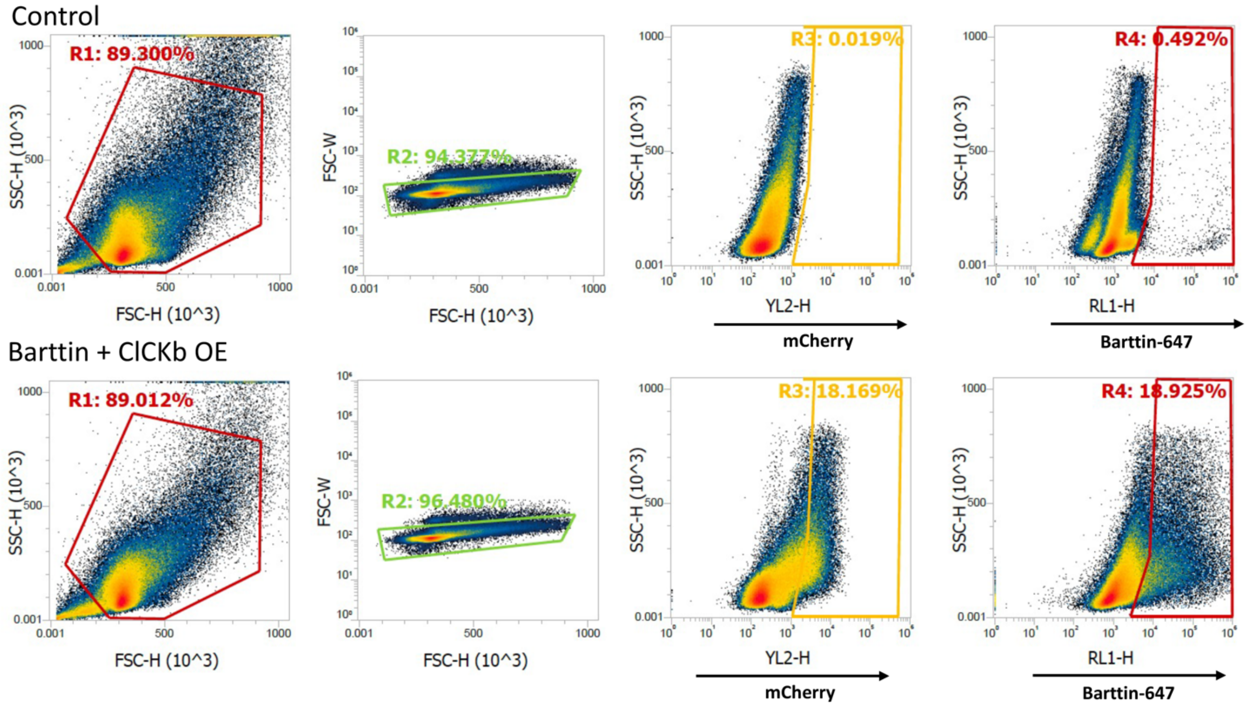

B Figure 8C Example Plots

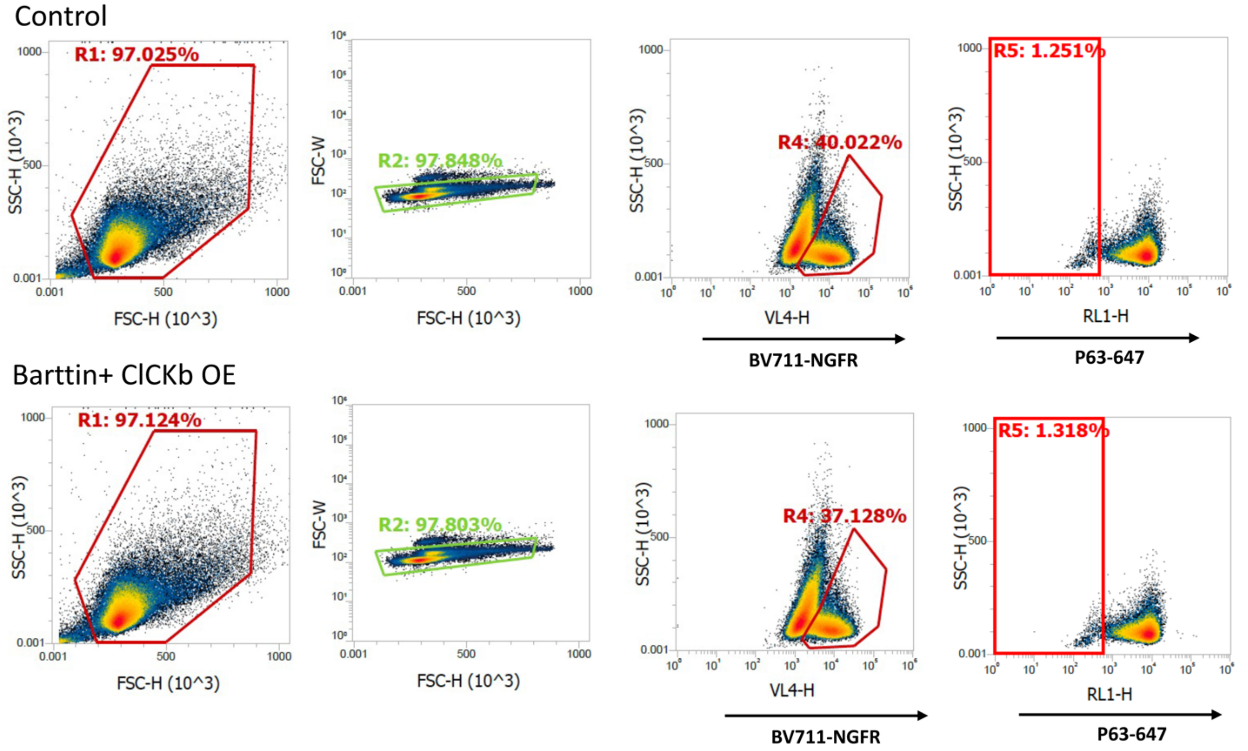

Supplemental Figure 4 Example flow cytometry analysis for Figure 8, B and C. Epithelia were transduced with an adenovirus vector expressing either GFP or a vector encoding barttin, ClC-Kb, and mCherry (Barttin + ClC-Kb OE) to add basolateral $Cl^–$ channels to non-ionocyte airway cells. Cells were gated by light intensities detected by a flow cytometer. **(A)** Example **Figure 8B**-type experiments. The gating scheme is shown from left to right. Side scatter height (SSC-H) vs. forward scatter height (FSC-H) was used to select for large and granular cells. FSC-width (FSC-W) vs. FSC-H was used to exclude cellular debris. Within this gated population, cells were sorted for mCherry (cells expressing the viral vector) or barttin. **(B)** Example **Figure 8B**-type experiment. The first 2 gates are as described for panel **A**. Additionally, cells were gated for NGFR (neuronal growth factor receptor) expression, which is on basal cells and ionocytes, then p63 was used to separate basal cells ($p63^+$) from ionocytes ($p63^–$).

Supplemental Figure 5

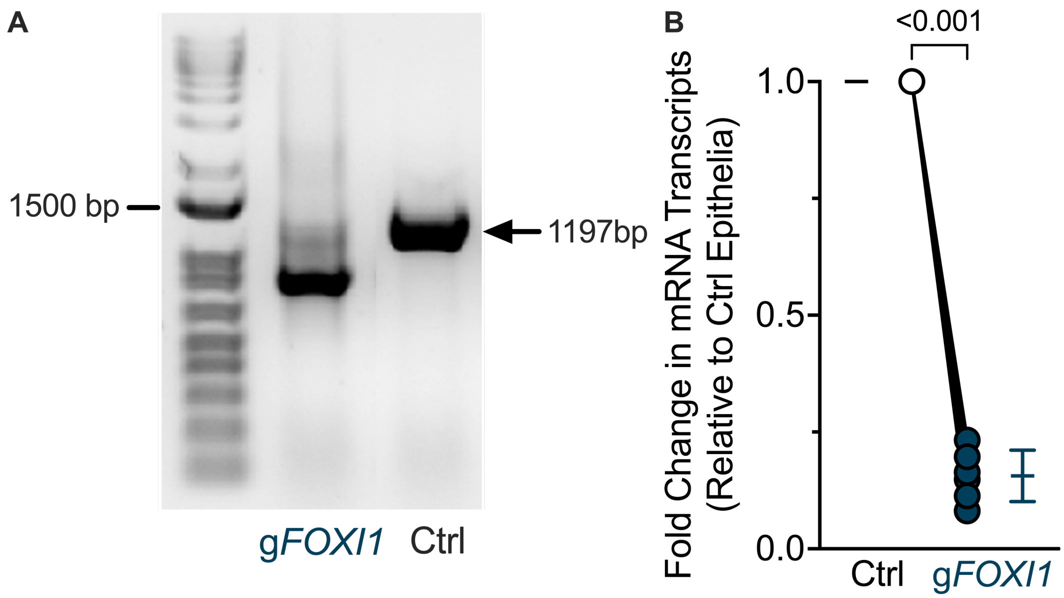

Supplemental Figure 5 Controls for *FOXI1* gene disruption. (A) Genomic PCR of human airway cells electroporated with and without g*FOXI1*. **(B)** RT-qPCR of *FOXI1* transcript levels in human airway cells electroporated with and without g*FOXI1*. Many g*FOXI1* samples did not show amplification and a threshold cycle of 40 was used for quantification. $n = 6$ human donors. Data points connected by a line represent paired experiments from a single human donor, graph depicts mean ± standard deviation, and the P value obtained from a paired two-sided Student's t test is presented within the figure.

Supplemental Figure 6

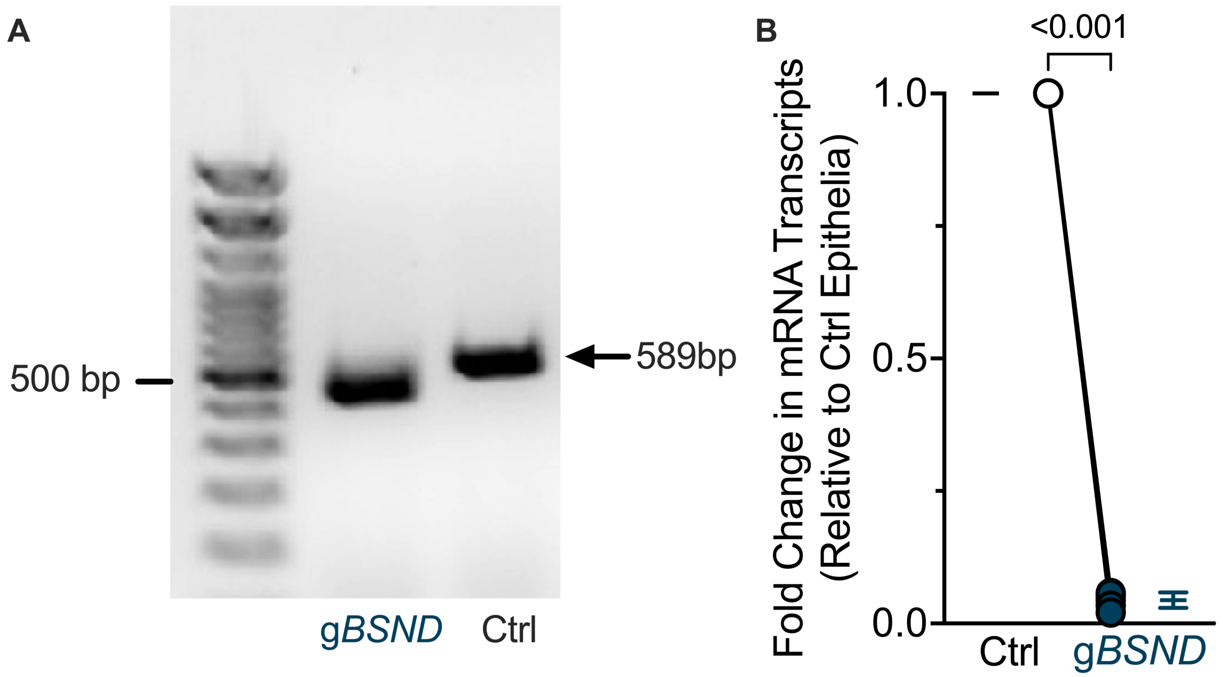

Supplemental Figure 6 Controls for *BSND* gene disruption. (A) Genomic PCR of human airway cells electroporated with and without g*BSND*. **(B)** RT-qPCR of *BSND* transcript levels in human airway cells electroporated with and without g*BSND*. Many g*BSND* samples did not show amplification and a threshold cycle of 40 was used for quantification. n = 6 human donors. Data points connected by a line represent paired experiments from a single human donor, graph depicts mean ± standard deviation, and the P value obtained from a paired two-sided Student's t test is presented within the figure.
